# Occupations in the European Labour Market During the COVID-19 Pandemic

**DOI:** 10.1007/s10272-022-1040-y

**Published:** 2022-04-08

**Authors:** Sara Flisi, Giulia Santangelo

**Affiliations:** 1 Joint Research Centre - Unit I.1, European Commission, Via E. Fermi 2749, TP 267, I-21027 Ispra (VA), Italy; 2 Joint Research Centre, European Commission, Via E. Fermi 2749, TP 267, I-21027 Ispra (VA), Italy

**Keywords:** J01, J21, J40, J63, J82

## Abstract

In order to capture the consequences of the COVID-19 pandemic on the labour market, several aspects need to be taken into account. First, containment measures put in place in member states at different times and with different levels of severity determined the interruption of several economic activities that were considered non-essential. Second, different occupations require varying degrees of physical proximity and social interaction to be carried out; this implies that they can be considered more or less teleworkable, and affected by different levels of epidemiological risk of contagion. This paper shows the labour market impact of the pandemic on different categories of workers in the EU. Occupations are distinguished by three main characteristics: whether they are critical or non-critical, their level of technical teleworkability and the level of social interaction required in the job. We show that the impact of the COVID-19 pandemic on the labour market has been heterogeneous across occupations and that all three dimensions are relevant to determine whether and to what extent the occupations were affected by the pandemic.

The COVID-19 pandemic revealed its unprecedented nature from the outset. From March to November 2020, over 10 million people in Europe had already been infected, with numbers continuing to rise rapidly (OECD and European Union, 2020), reaching nearly 240 million cases at the global level by mid-October 2021 (Johns Hopkins Coronavirus Resource Center, 2021). Countries implemented a wide range of policy responses, such as lockdown measures and stay-at-home orders to contain and mitigate the spread of the virus. Inevitably, this also impacted the labour market.

During the course of 2020, with official data on the real impact of the pandemic still missing, a number of studies attempted to simulate the possible labour market effect on workers by relying on different classifications of jobs, applied to pre-pandemic data.

Among these, a first group of investigations focused on levels of occupational exposure to contagion. Basso et al. (2020) classified occupations according to epidemiological risk of contagion for the US, the EU and the UK; Lewandowski (2020) developed a methodology to measure country-specific levels of occupational exposure to contagion among workers in the same occupations for 26 European countries using the European Working Conditions Survey (EWCS). Focusing on Italy, and considering the first waves of lockdowns, Barbieri et al. (2020) classified the occupations according to the workers' disease exposure, physical proximity and possibility to work remotely, mapping them into sectoral indices, based on the Italian Sample Survey on Professions (ICP).

A second group studied the potential for business continuity throughout containment measures, thanks in part to the possibility of working from home. Dingel and Neiman (2020) were the first to develop a classification for all occupations according to the feasibility of working from home, which was followed extensively in the successive literature. They based their classification on data from the US Occupational Information Network (O*NET) Work Context Questionnaire and merged this classification with occupational employment counts in the US. For the EU, Sostero et al. (2020), following a task approach, defined teleworkability indicators of physical and social interaction based on the tasks of specific occupations. They exploited the information from both the ICP and EWCS and quantified the fraction of employees in teleworkable occupa-

* The authors would like to thank Alessia Fulvimari and Tim van Rie for suggestions that significantly improved the paper and previous versions of the analysis. The scientific output expressed does not imply a policy position of the European Commission. Neither the European Commission nor any person acting on behalf of the Commission is responsible for the use which might be made of this publication.

**Sara Flisi**, European Commission, Joint Research Centre, Ispra, Italy.

tions across EU countries, sectors and socio-economic profiles, using 2018 data. Fana et al. (2020) assessed the potential impact of the early 2020 COVID-19 confinement measures on EU labour markets, analysing the restrictions on economic activity imposed in Italy, Spain and Germany. Finally, for country-specific studies, Cetrulo et al. (2020) described the Italian occupational structure, quantifying the jobs that can be done from home based on the ICP, while Crowley and Doran (2020) used O*NET data to construct social distancing and remote working potential indices for the Irish labour market.

Physical contact, proximity to others and digital skills required were also used by Pouliakas and Branka (2020) to construct a social distancing risk index for employment in the 27 EU member states, building on the first wave of Cedefop's European skills and jobs survey from 2014.

An additional branch of the recent literature exploited real-time data from surveys run right after the outbreak of the pandemic to describe its labour market outcomes, revealing large cross-country differences (see, among others, Galasso and Foucault (2020) for twelve countries across Europe and the US; and Adams-Prassl et al. (2020) for the UK, US and Germany). Building on the latter, Adams-Prassl et al. (2022) also documented the variation within and across occupations and industries of tasks workers can do from home.

Finally, looking at the potential impact of the pandemic on future employment forecasts, Livanos and Ravanos (2021) assessed possible future short- and long-term employment loss due to the pandemic, according to the potential of a job to be carried out remotely or substituted by automation, using Cedefop employment and skills forecasts.

To our knowledge, however, no study followed up on these first analyses, using official data on the period following the start of the pandemic to check whether these classifications indeed helped capture its real impact on different categories of workers.

This paper contributes to the literature by providing evidence on the real labour market impact of the pandemic on different categories of workers in the EU. We develop a taxonomy to identify categories of workers who might have been more or less severely hit by the COVID-19 pandemic. We consider three main characteristics of jobs, namely (i) whether they are critical or non-critical, (ii) their level of technical teleworkability and (iii) the level of social interaction required in the job. We build indices on occupational groups defined at the International Standard Classification of Occupations (ISCO) 3-digit level and analyse the employment evolution of occupations in these groups between 2019 and 2020 by using data from the European Union Labour Force Survey (EU-LFS). The next section illustrates the methodology and classification adopted, as well as the data source used and depicts the results of our descriptive analysis, followed by our conclusions.

## Methodology and data

To provide a complete picture of the legacy of COVID-19 on labour market outcomes and of changes in working conditions induced by the pandemic, one has to consider different aspects.

First, containment and lockdown measures put in place at the national level at different times and with varying levels of severity determined the interruption of several economic activities that were considered non-essential or more at risk due to the higher threat of contagion. During the first lockdown phase, several countries applied a strict dichotomous categorisation of sectors into essential and non-essential ones, where the latter were formally shut down, unless they could operate remotely. Such provisions were often later relaxed to allow non-essential activities to re-open, under the condition that stringent health and safety requirements were met. From the last months of 2020, in a vast majority of countries, only a few selected sectors were officially shut down (e.g. museums, cinemas, gyms), or allowed to operate with strong limitations (e.g. restaurants and cafés).

Second, different occupations require varying degrees of physical proximity and social interaction; they are therefore subject to different levels of epidemiological risk of contagion and imply different levels of teleworkability. Jobs requiring tasks which do not necessitate physical and social interaction, and can be performed entirely from home, can be considered teleworkable, and therefore "safe" in terms of disease exposure. Conversely, occupations that require interactions can be ranked according to the physical proximity entailed in their execution and classified as more or less "unsafe" due to the different levels of the contagion risk they entail. Teleworkability can also favour business continuity even in lockdown periods, hence reducing potential risks of job disruption.

### Critical occupations

The distinction between essential and non-essential – or, more broadly speaking, between "shut down" and "not shut down" – economic sectors is done in the literature mostly by looking at national decrees on lockdown and containment measures adopted by single countries during the COVID-19 pandemic. Most of the European studies (Fana et al., 2020; Barbieri et al., 2020) indeed refer to the lockdowns adopted in selected EU countries between March and April 2020, when the containment measures were the strictest. However, for the purposes of an EU-wide analysis, the definition of jobs in essential and non-essential economic sectors is especially challenging. First, this distinction strictly depends on containment and lockdown measures implemented in each single country, and EU-wide generalisations of national measures are hard to apply. Secondly, even when trying to apply national rules, the identification of economic sectors that were considered essential or non-essential, and hence shut down or not, was often done at a very detailed level of economic activity, which is hard to reproduce using official cross-country statistics.[1]

To overcome these limitations, for the purpose of the analysis presented in this paper, we identify "critical occupations" based on the categorisation provided by the Communication from the Commission on Guidelines concerning the exercise of the free movement of workers during the COVID-19 outbreak (European Commission, 2020a). This Communication defines a list of "key workers" that should be allowed to move across borders "because they exercise critical occupations by performing activities related to essential services", which they should be able to do "without undue hindrance". This categorisation (even though originally devised to allow cross-country mobility) provides a distinction between workers who were most likely allowed to continue working even under the strictest containment measures, and those who were not, unless working from home.[2] Our approach is similar to the one adopted by Fasani and Mazza (2020) and OECD (2020), which also resort to the Communication to define key workers. This dichotomous variable capturing critical occupations represents the first dimension of our taxonomy.[3]

### Teleworkability and social interaction

The second component of our taxonomy considers the task content of occupations. Studies focusing on the features of the job to establish teleworkability, but also epidemiological risk for workers, normally rely on data sources capturing exactly this task content. Based on this information, each occupation can be classified according to different possible indices, such as physical proximity, social interaction or teleworkability.

For this part of the analysis, we rely on the indices of technical teleworkability and social interaction developed by Sostero et al. (2020), which allow the identification of jobs that can be done from home, and with a certain level of quality. The teleworkability index identifies jobs that are technically teleworkable or not, based on the amount of physical interaction involved in a range of physical tasks.[4] The complementary index of social interaction[5] serves as a qualification of the assessment of technical teleworkability; as a matter of fact, despite the technical feasibility of carrying out a job remotely if needed, some occupations involve a high degree of social interaction; the index is based on the assumption that a higher relevance of social interaction tasks implies a lower quality of the service provided when teleworking (e.g. for teachers).[6] Based on these two indices, occupations in the EU can be classified according to their level of technical teleworkability and social interaction required; this distinction allows identifying jobs that might have been more at risk of job disruption during the pandemic.

There are a number of reasons to rely on these indicators from Sostero et al. (2020) rather than one of the many other similar classifications developed during the first months of the pandemic.

First of all, this study has the advantage of being based on data that is specific to the European context, i.e. the Italian ICP. As mentioned above, many of the first studies on work

1 As an example, the initial identification of essential activities in Italian decrees relied on classifications of sectors as detailed as at the 5- or even 6-digit level of NACE, i.e. the Statistical classification of economic activities in the European Community. EU-LFS microdata only provide information on the economic sector of jobs at the NACE 1-digit level.

2 It should also be noted that since March 2020, the containment measures have been subject to several changes. As mentioned above, the initial strict lockdowns implemented in most of the countries were followed by a strong relaxation of these measures; and with the second wave of the pandemic, only a few selected sectors were officially shut down or allowed to operate with strong limitations, therefore abandoning the initial distinction of essential vs non-essential jobs. Nevertheless, we think that this EU classification can help capture a relevant dimension in the occupations, not only in the first months of the pandemic.

3 Starting from the list provided in this Communication, we identified workers exercising critical occupations as those working in the following ISCO 2- and 3-digit categories: 213 life science professionals; 214 engineering professionals (excluding electrotechnology); 215 electrotechnology engineers; 22 health professionals; 23 teaching professionals; 25 information and communications technology professionals; 31 science and engineering associate professionals; 32 health associate professionals (except 323 traditional and complementary medicine associate professionals); 35 information and communications technicians; 53 personal care workers; 61 market-oriented skilled agricultural workers; 62 market-oriented skilled forestry, fishery and hunting workers; 63 subsistence farmers, fishers, hunters and gatherers; 751 food processing and related trades workers; 816 food and related products machine operators; 83 drivers and mobile plant operators; 91 cleaners and helpers; 92 agricultural, forestry and fishery labourers; 93 labourers in mining, construction, manufacturing and transport; 96 refuse workers and other elementary workers.

4 In particular, the technical teleworkability index considers: manual dexterity; finger dexterity; performing general physical activities; handling and moving objects; inspecting equipment, structures, or material; operating vehicles, mechanized devices, or equipment; lifting or moving people.

5 Social interaction tasks include: selling or influencing others; training and teaching others; assisting and caring for others; performing for or working directly with the public; coordinate the work and tasks of others.

6 While originally conceived to proxy for the loss of quality in a technically teleworkable job, the relevance of social interaction can be an important aspect also within non-teleworkable ones, as it can capture aspects such as the risk of exposure to contagion that a worker can run, as well as a risk of undergoing limitations in business continuity due to containment measures. Such distinction is therefore applied for both teleworkable and non-teleworkable occupations.

from home relied on classifications based on the US O*NET (Dingel and Neiman, 2020; Basso et al., 2020; Lewandowski, 2020; European Commission, 2020b); however, the use of this source – and the subsequent international crosswalk required to analyse EU labour markets – implies the rather strong assumption that the content of occupations in the US is similar to that of European jobs. The Italian ICP, instead, might be better able to capture the structure of European occupations (Barbieri et al., 2020; Cetrulo et al., 2020).[7]

Second, it allows a good level of granularity, since it provides technical teleworkability and social interaction indices that are computed at the ISCO 3-digit level, which is also the maximum level of disaggregation available in EU-LFS data.[8]

Finally, as explained by the authors, these indices are anchored to a task framework developed for occupational analysis (Fernández-Macías and Bisello, 2020) that provides a detailed justification for the items taken into account in the ICP survey.

## Data sources

We combine the critical occupations, technical teleworkability and social interaction indices to create a comprehensive classification of EU occupations, which we then apply to data from a Eurostat special extraction from the 2019 and 2020 EU-LFS. We do this by matching the categorisation of occupations to the jobs' ISCO code available in the EU-LFS, to investigate the employment evolution of occupations based on these three characteristics of jobs. The extractions provided quarterly data, allowing for comparisons in the evolution of employment in specific categories of occupations between the same quarter of different years.

## Results

Data from the EU-LFS enable an investigation into the drop in employment registered in the EU. The second quarter of 2020 was the most strongly affected by the pandemic, with widespread lockdowns implemented in several countries. After a period of relative improvement, the last quarter of the year was again severely impacted,

Figure 1

**Employment change by occupational group, EU26, 2019/2020**

in %

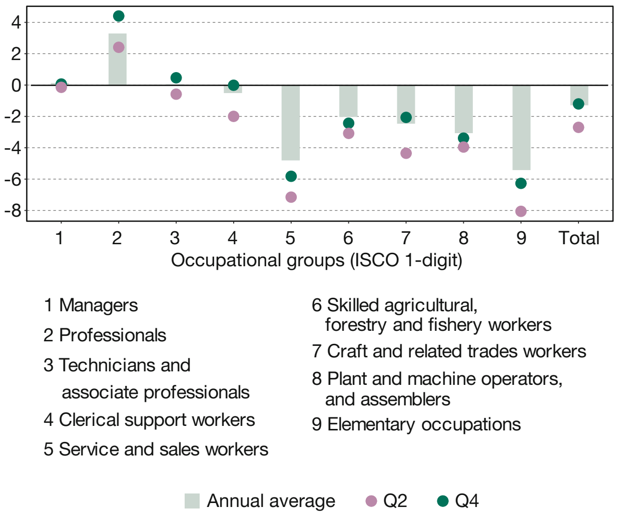

Notes: Data refer to the age group 20-64. Armed forces are not taken into account in the analysis. Germany is excluded from the analysis due to a break in the time series. The figure shows the employment change in Q2/Q4 of 2020 (and the annual average) compared to the corresponding period in 2019.

Source: Authors' calculations based on a Eurostat special extraction from EU-LFS data.

with a second outbreak hitting many EU countries. For this reason, we analyse changes in employment for these two quarters; we also show the annual average, to summarise the impact throughout the year.

Overall, employment in the EU[9] decreased by less than 3% between Q2 of 2019 and the same quarter of 2020, and by 1.2% in Q4. This relatively limited overall decrease hides considerable differences between categories of workers, and in particular between different occupational groups. Figure 1 shows the employment change by ISCO 1-digit occupations.

Between 2019 and 2020, most occupational groups saw a decline in the level of employment, both in the second and fourth quarters of the year. This is especially the case for low- and medium-skilled occupations (ISCO 4-9), with a stronger decrease in Q2 than Q4. Elementary occupations and service and sales workers are the categories with the highest employment drops. High-skilled occupa-

7 As already mentioned, the indicators by Sostero et al. (2020) partly rely on another data source, the EWCS.

8 Unlike O*NET and the Italian ICP, the EWCS allows for measuring cross-country differences in the nature of work of comparable occupations (Lewandowski, 2020); however, it provides a much lower level of disaggregation when analysing occupations (ISCO 2-digits instead of e.g. 5-digit classification in ICP). The apparent advantage of cross-country comparison is therefore likely undermined by the lower precision in the analysis of the individual occupations, since the most detailed level available is already an aggregation of several occupations, possibly very diverse in nature. For this reason, we prefer to rely on a classification based mostly on more granular ICP data.

9 We show in this paper figures referring to EU26, i.e. EU27 member states except Germany. This is because of changes in the survey methodology for Germany in the LFS, which led to a break in German data in 2020. Since estimates for 2020 cannot be compared directly with those of previous years, we decided to discard the country from the analysis.

Figure 2
**Distribution of employment by occupational characteristics, EU27, 2019**

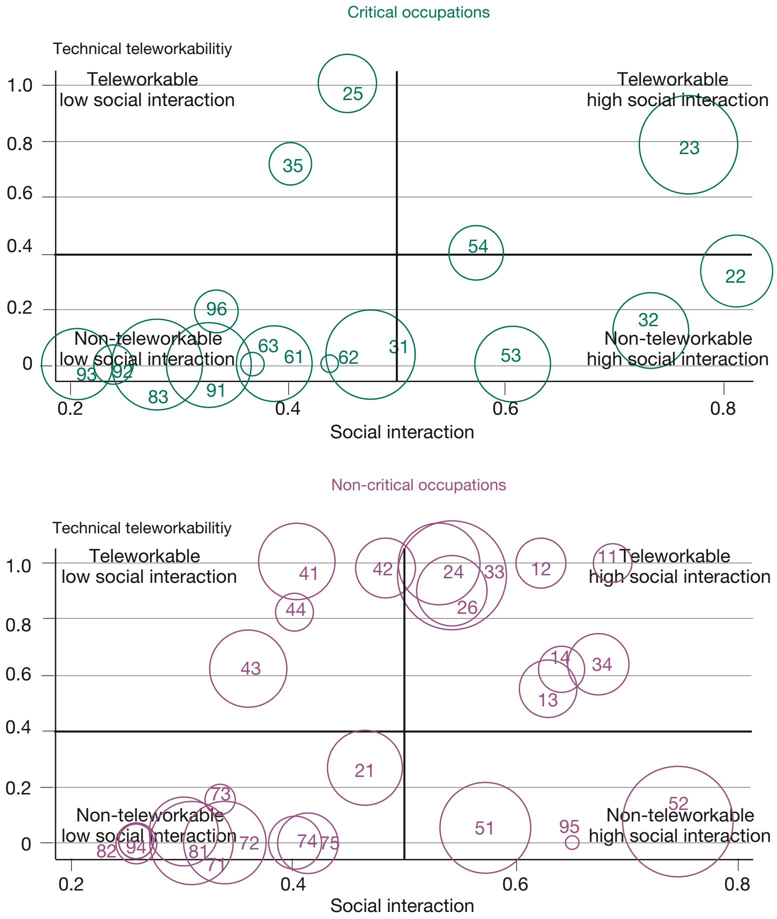

Occupational groups (ISCO 2-digits)

11 Chief executives, senior officials and legislators
12 Administrative and commercial managers
13 Production and specialised services managers
14 Hospitality, retail and other services managers
21 Science and engineering professionals
22 Health professionals
23 Teaching professionals
24 Business and administration professionals
25 Information and communications technology professionals
26 Legal, social and cultural professionals
31 Science and engineering associate professionals
32 Health associate professionals
33 Business and administration associate professionals
34 Legal, social, cultural and related associate professionals
35 Information and communications technicians
41 General and keyboard clerks
42 Customer services clerks
43 Numerical and material recording clerks
44 Other clerical support workers
51 Personal service workers
52 Sales workers
53 Personal care workers
54 Protective services workers
61 Market-oriented skilled agricultural workers
62 Market-oriented skilled forestry, fishery and hunting workers
63 Subsistence farmers, fishers, hunters and gatherers
71 Building and related trades workers, excluding electricians
72 Metal, machinery and related trades workers
73 Handicraft and printing workers
74 Electrical and electronic trades workers
75 Food processing, wood working, garment and other craft and related trades workers
81 Stationary plant and machine operators
82 Assemblers
83 Drivers and mobile plant operators
91 Cleaners and helpers
92 Agricultural, forestry and fishery labourers
93 Labourers in mining, construction, manufacturing and transport
94 Food preparation assistants
95 Street and related sales and service workers
96 Refuse workers and other elementary workers

Notes: The top panel corresponds to critical occupations and the bottom one to non-critical occupations, according to the definition provided above. Within each panel, the chart is divided into four quarters based on whether the teleworkability and social interaction indices are above or below a certain threshold. The size of the bubble represents the size of employment in the corresponding occupation in 2019. Data refer to the age group 20-64. Armed forces are not taken into account in the analysis.

Source: Authors' calculations based on a Eurostat special extraction from EU-LFS data.

tions, on the other hand, did not register such a decline in employment levels; professionals even saw an increase in both Q2 and Q4, while technicians and associate professionals, after an initial drop in Q2, recovered in the fourth quarter of 2020.

Various features of occupations might explain these patterns. As mentioned above, we identify three dimensions along which occupations might differ, i.e. whether they are critical or not; their level of technical teleworkability; and the level of social interaction required by the job. The latter two indices are expressed on a scale from 0 to 1.

Figure 2 shows a picture of the distribution of employment along these dimensions in the year before the outbreak of the pandemic.[10] Each occupation is represented by a circle whose size is proportional to the number of individuals employed in that occupation in 2019.

The top panel is clearly less densely populated than the bottom one, as few occupations can be considered critical. However, what we notice is that in both panels, many

10 For the sake of clarity, the figure shows occupations at the ISCO 2-digit level. For the purposes of this figure only, the technical teleworkability and social interaction indices, as well as the critical occupations indicator, were aggregated from the ISCO 3-digit to the 2-digit level based on the relative weight of employment in 3-digit occupations in each EU member state in 2019. This procedure is in line with the one used by Sostero et al. (2020) to aggregate from 5-digit Codici Professionali into 3-digit ISCO categories.

circles are concentrated around low values of the technical teleworkability index, suggesting that a high share of occupations cannot be performed remotely at all. This is especially true for critical occupations.

To investigate more in depth the impact of COVID-19 on labour market outcomes, we therefore show the employment evolution of occupational categories defined in terms of these dimensions. Beyond distinguishing between critical and non-critical occupation, we also apply a dichotomous definition of teleworkability and social interaction, as illustrated in Figure 2. In detail, we build on the definitions adopted by Sostero et al. (2020), where an occupation is technically teleworkable if its value of the technical teleworkability index is higher than 0.4, and the level of social interaction required in the job is low/high depending on whether the social interaction index is lower/higher than 0.5. Based on these thresholds, we transform the two continuous indices into binary variables. The two variables are then interacted to create four categories, consisting of occupations that are: (i) non-teleworkable and requiring high social interaction (health professionals; health associate professionals; care, service and sales workers); (ii) non-teleworkable, but requiring low social interaction (such as skilled agricultural, forestry and fishery workers; craft and related trade workers; plant and machine operators and assemblers; most elementary occupations); (iii) teleworkable with high social interaction (e.g. managers; teaching professionals; business, administration, legal, social and cultural professionals and associated professionals); and (iv) teleworkable with low social interaction (such as clerical support workers and ICT professionals). Within each of the above four categories, we further distinguish between critical and non-critical occupations, leading to eight categories in total.

As shown in Figure 3, teleworkability seems to be a main determinant of the employment changes during the pandemic year. While employment in non-teleworkable occupations experienced sharp decreases between 2019 and 2020, employment in teleworkable ones remained stable, and in some cases even increased. On the other hand, less clear patterns emerge for social interaction. In each of the four occupational categories identified, critical occupations registered a better performance than non-critical ones.

Critical, teleworkable occupations are indeed the ones with the most positive developments in terms of employment levels. These occupations saw an increase in employment throughout the whole year. This growth was higher for jobs requiring low social interaction (up to 10% in Q4 2020), and was mostly driven by higher levels of employment among information and communications technology professionals (such as software and applications

Figure 3

**Employment change by occupational category, EU26, 2019/2020**

in %

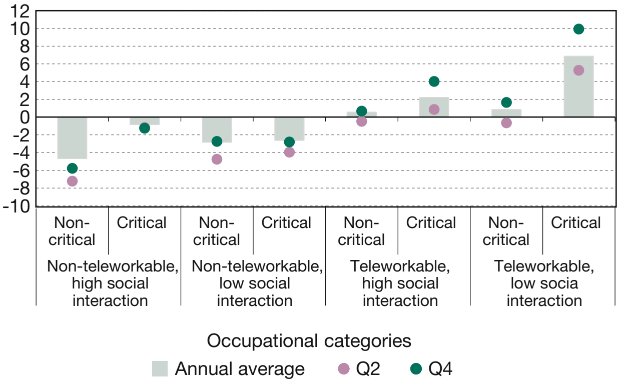

Notes: Data refer to the age group 20-64. Armed forces are not taken into account in the analysis. Germany is excluded from the analysis due to a break in the time series. The figure shows the employment change in Q2/Q4 of 2020 (and the annual average) compared to the corresponding period in 2019.

Source: Authors' calculations based on a Eurostat special extraction from EU-LFS data.

developers and analysts as well as database and network professionals). Activities in such occupations were essential and therefore allowed to continue operating even under the strictest containment measures; moreover, they could easily be performed remotely and were likely highly requested due to the widespread use of telework during the pandemic. Critical, teleworkable jobs requiring high social interaction, on the other hand, registered only a small increase. This is likely because these occupations can be performed remotely from a technical point of view, but suffer a loss in the quality of the work done. This is the case, for example, for teaching professionals. Employment in teleworkable but non-critical occupations (such as clerical support workers) remained rather stable in 2020.

Unlike teleworkable occupations, non-teleworkable ones suffered a clear decline in employment. Within this group, the smallest decline was registered among critical occupations requiring high social interaction; these include, among others, health professionals and associate professionals (such as doctors and nurses, occupations which were at the forefront of the pandemic, and saw even a small increase in employment), childcare workers and personal care workers. The highest employment drop was experienced in non-critical occupations among the non-teleworkable ones that require high social interaction. This was the case, for instance, for sales workers, who registered a decrease close to 3% in the pandemic year, and for waiters and bartenders, who experienced a decrease of over 15%.

Overall, teleworkability seems to have provided the strongest protection against job losses during the pandemic, and especially during the lockdowns. Among teleworkable occupations, critical occupations even experienced an increase in employment, especially those requiring limited social interaction. Non-teleworkable, non-critical jobs, particularly those that require high social interaction, underwent the strongest declines in employment.

## Conclusions

In this paper we show the labour market impact of the pandemic on different categories of workers in the EU. We distinguish occupations based on three main characteristics of jobs, namely (i) whether they are critical or non-critical; (ii) their level of technical teleworkability; and (iii) the level of social interaction required in the job. We show that all three dimensions are relevant to determine whether and to what extent the occupations were affected by the pandemic.

Based on 2019 and 2020 data at the EU level, our analysis contributes to showing that the impact of the COVID-19 pandemic on the labour market has been heterogeneous. While employment in non-teleworkable occupations decreased significantly, some teleworkable occupations registered a considerable increase in employment.

Among non-teleworkable occupations, the decline was less pronounced for critical jobs requiring high social interaction, such as doctors and nurses. Among teleworkable jobs, employment in critical occupations increased, especially among those requiring low social interaction (such as ICT professionals and technicians).

As highlighted in European Commission (2021), this type of evidence can help shed light on the labour market disparities exacerbated by the pandemic, hence contributing to the public debate on employment and social development issues, and on the measures that can promote the economic recovery at the EU level. Member states, as well as the EU, put forward a very strong policy response throughout 2020, offering unprecedented levels of assistance. The packages of measures implemented ranged from more accessible unemployment benefits, to expanded paid sick leave, to more widely available and more generous short-time work schemes especially for small and medium-sized firms, as well as exceptional income support measures.

Recent history has shown that additional waves of the COVID-19 pandemic and subsequent containment measures with targeted restrictions cannot yet be ruled out. Learning from the experience of the last two years, the evidence we provide in this paper can help shape the design of both social distancing restrictions and support schemes in a timely and targeted way, with the goal of limiting the damages and sustaining the categories that are more in need and more at risk of being heavily affected.
